# Neurobiology of SARS-CoV-2 interactions with the peripheral nervous system: implications for COVID-19 and pain

**DOI:** 10.1097/PR9.0000000000000885

**Published:** 2021-01-07

**Authors:** Amelia J. McFarland, Muhammad S. Yousuf, Stephanie Shiers, Theodore J. Price

**Affiliations:** Department of Neuroscience and Center for Advanced Pain Studies, University of Texas at Dallas, Richardson, TX, USA

**Keywords:** COVID-19, SARS-CoV-2, Neuropathy, Nociceptor

## Abstract

SARS-CoV-2 is a novel coronavirus that infects cells through the angiotensin-converting enzyme 2 receptor, aided by proteases that prime the spike protein of the virus to enhance cellular entry. Neuropilin 1 and 2 (NRP1 and NRP2) act as additional viral entry factors. SARS-CoV-2 infection causes COVID-19 disease. There is now strong evidence for neurological impacts of COVID-19, with pain as an important symptom, both in the acute phase of the disease and at later stages that are colloquially referred to as “long COVID.” In this narrative review, we discuss how COVID-19 may interact with the peripheral nervous system to cause pain in the early and late stages of the disease. We begin with a review of the state of the science on how viruses cause pain through direct and indirect interactions with nociceptors. We then cover what we currently know about how the unique cytokine profiles of moderate and severe COVID-19 may drive plasticity in nociceptors to promote pain and worsen existing pain states. Finally, we review evidence for direct infection of nociceptors by SARS-CoV-2 and the implications of this potential neurotropism. The state of the science points to multiple potential mechanisms through which COVID-19 could induce changes in nociceptor excitability that would be expected to promote pain, induce neuropathies, and worsen existing pain states.

## Abstract 2

We review what is currently known about how the SARS-CoV-2 may interact with nociceptors to enhance pain and potentially cause or worsen neuropathies in COVID-19.

## 1. Introduction

A growing area of study in the area of host–microbe interactions is examining how microbes interact directly with the sensory nervous system. Because pain is a primary symptom of many types of infection and a lack of pain can help microbes to evade early detection, investigators have focused on microbe interactions with nociceptors in the dorsal root ganglia (DRG) and trigeminal ganglia (TG). These studies have revealed insights into how bacterial products interact directly with nociceptors to either promote or inhibit pain.^17^ The *Staphylococcus aureus* bacteria produce N-formylated peptides and α-haemolysin, both of which act directly on nociceptors to cause pain during *Staphylococcus* infections.^16^ Another example is in tuberculosis where the *Mycobacterium tuberculosis* produces sulfolipid-1 that acts on transient receptor potential vanilloid type 1 (TRPV1)-positive neurons to evoke cough.^73^ On the other end of this spectrum, nociceptors can also play a key role in reducing bacterial pathogenesis, such as in the case of *Salmonella enterica* where calcitonin gene-related peptide released from TRPV1-positive neurons protects against infection after the detection of the pathogen by nociceptor nerve endings in the gut.^42^ These studies demonstrate the intricacies of how bacteria can interact with nociceptors to promote pain, influence infection severity, or play a key role in promoting the spread of disease.

Great strides have been made in understanding how bacteria and nociceptors interact, but advances in how viruses affect nociceptors lag behind. This is somewhat surprising for 2 reasons. First, until very recently, postherpetic neuralgia caused by the varicella-zoster virus was one of the most common forms of neuropathic pain. In this case, the virus sits dormant in DRG or TG neurons after chickenpox until it is reactivated causing an acute case of shingles that is often followed by the persistent neuropathic pain syndrome, postherpetic neuralgia. Although recent advances have been made in understanding basic mechanisms causing pain in postherpetic neuralgia,^39,81^ we may eliminate this neuropathic disease through vaccination before we fully grasp its neurobiology. Second, it is well known that one of the earliest signs of viral infection is body-wide aches and pain that often begin before the fever starts. Recent studies have started to unravel how this happens,^6,32^ but it is still an area of active investigation with many unaddressed questions.

This lack of knowledge presents a quandary for the COVID-19 pandemic. It is now clear that there are neurological effects of SARS-CoV-2 at both the early and later stages of disease progression, including in “long COVID,” but there is no existing precedent for how coronaviruses might interact with DRG or TG neurons. In this review, we will cover several possibilities and discuss their implications for COVID-19. We will start with experimental insight into how viruses might induce pain through 2 pathways, by type I interferons^6^ or by induction of indoleamine 2,3 dioxygenase (IDO1), and subsequent production of kynurenine.^32^ We will then discuss what we currently know about cytokine dysregulation in COVID-19 and how that unique pattern of immune reaction^4,55^ may drive interactions with nociceptors that would be expected to promote pain and potentially worsen existing pain problems. Finally, we will discuss the evidence for neurotropism of SARS-CoV-2 and the possibility for direct viral infection of nociceptors through angiotensin-converting enzyme 2 (ACE2) receptors, proteases such as furin,^18^ and additional host entry receptors neuropilin 1 and 2 (NRP1 and NRP2).^13,19^ We think that these insights can inform how clinicians approach dealing with persistent pain and/or neuropathies in patients who suffer or have suffered from COVID-19.

## 2. Neurobiology of virus-induced pain

Unlike bacteria, viruses have small genomes that encode relatively few proteins. The SARS-CoV-2 genome has 14 open reading frames encoding 27 proteins, including the spike protein that is known to bind ACE2.^95^ The spike protein can also be cleaved by furin, priming the protein to bind for NRP1 and NRP2.^13,19^ It is likely that the presence of ACE2, furin, and neuropilins increases the probability that a cell can be invaded by SARS-CoV-2. Although this provides some diversity in mechanisms through which viruses can act on neuronal receptors, many viral proteins do not have clear targets in host cells. Moreover, individual viruses use divergent receptor mechanisms to enter host cells (ACE2 for SARS-CoV-2^100^; CCR5 for HIV^57^; and nicotinic acetylcholine receptors, p75 NGF receptors, and neuronal cell adhesion molecule for rabies^41^), and different viruses often infect distinct host cell types. Therefore, it is unlikely that the body-wide aches and pain—one of the earliest signs of many viral infections—are driven by viral proteins interacting directly with nociceptors because these mechanisms are not conserved across viruses. An early feature of viral infection is the production of nucleic acids that are found in the extracellular space. SARS-CoV-2 is a positive sense RNA virus so produces extracellular structured single-stranded RNA.^24^ These extracellular nucleic acids (either RNA or DNA) are signs of infection or cellular damage and thus induce a strong immune response. Extracellular RNA, which is a clear sign of infection by RNA viruses, act on toll-like receptor 3 that turns on production of type I interferons in host cells.^56^ The production and release of type I interferons is the first step in the host antiviral response.^69^

Because of this well-known role of type I interferons as the body's first response to viral infection, we recently tested the hypothesis that interferon α and β, the 2 type I interferons, might promote pain through a direct action on nociceptors. In mice, we found that the receptor complex for type I interferons is robustly expressed by nociceptors and injection of these interferons causes mechanical hypersensitivity in both male and female mice.^6^ In addition to these behavioral effects, type I interferon application to mouse nociceptors in vitro produced increased excitability consistent with the observed behavioral effects. Type I interferons induce signaling in host cells that protects them from viral replication. This involves induction of phosphorylation of elongation initiation factor 2α (eIF2α) that suppresses translation, blocking the ability of virus to produce the viral coat proteins they need for replication.^5^ Induction of eIF2α phosphorylation is known to promote pain in a number of pathological conditions associated with sensory neuropathy.^7,36,102^ To our surprise, we noted that type I interferons did not induce an eIF2α-mediated response in nociceptors. Rather, we observed induction of mitogen-activated protein kinase–interacting kinase (MNK1/2)-mediated eIF4E phosphorylation,^6^ a different pathway that promotes the synthesis of proteins from a subset of mRNAs that are associated with promoting pain.^58,61,65,66,75^ Pain-promoting behavioral effects of type I interferons were absent in mice lacking this MNK1/2-eIF4E signaling pathway. These findings support the conclusion that type I interferons promote virus-induced pain through an action on sensory neurons where these antiviral proteins induce a distinct signaling response that increases the excitability of nociceptors.^6^ Importantly, we did not find any evidence that extracellular viral RNA has a direct action on mouse nociceptors. This indicates that other cell types are needed to detect the presence of virus and produce type I interferons that then act on nociceptors.

**Figure 1.**
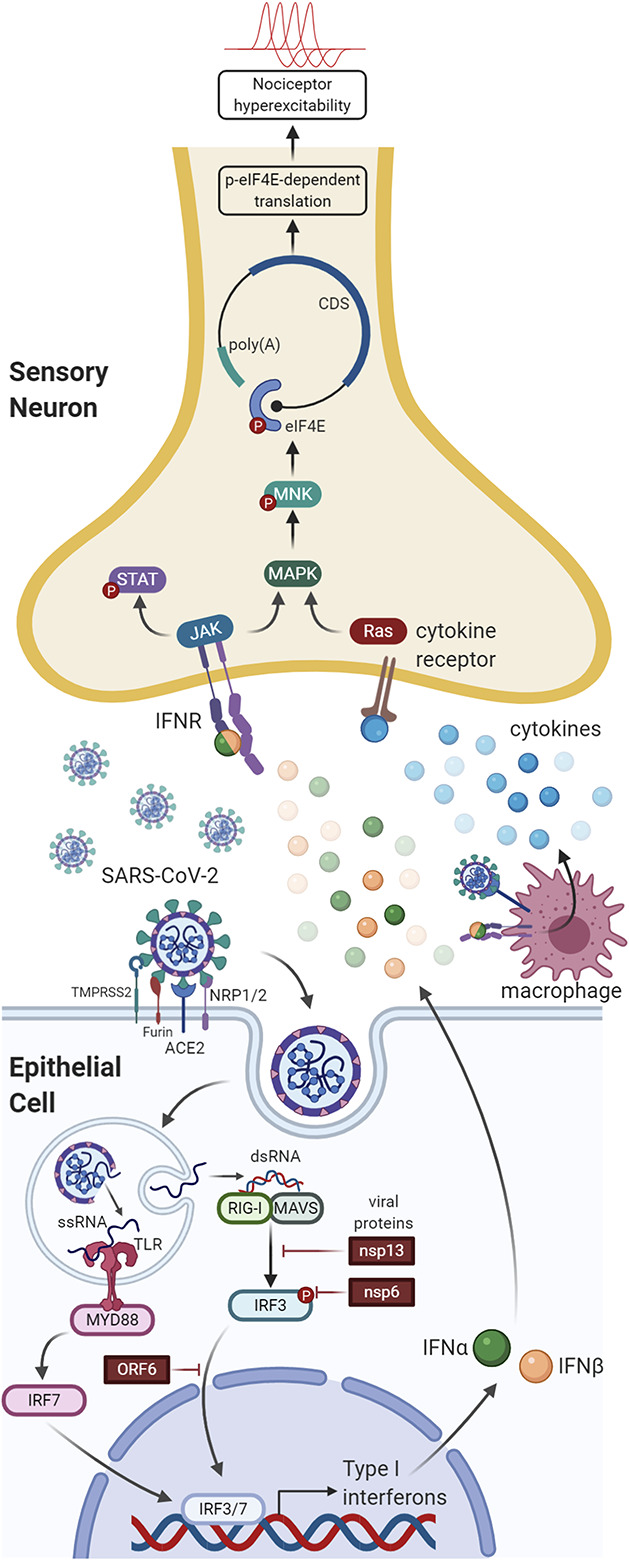
Cellular response to SARS-CoV-2 infection in the lung and its relationship to nociceptors. The cleavage of viral S protein by surface proteins such as TMPRSS2 and furin facilitates SARS-CoV-2 entry into lung epithelial cells through the ACE2 receptor. Neuropilin 1 and 2 (NRP1/2) act as additional viral entry factors. Viral RNA activates interferon regulatory factors (IRF3 and IRF7) that promote type I interferon (IFNα/β) production. The binding of type I IFNs to IFN receptors on pulmonary nociceptors is postulated to stimulate MNK-mediated eIF4E phosphorylation, resulting in nociceptor sensitization. When the viral load is high, viral proteins (NSP13, NSP6, and ORF6) suppress IFN production, contributing to evasion of type I IFN production and increasing the severity of COVID-19. ACE2, angiotensin-converting enzyme 2.

Based on this, one might hypothesize that type I interferons produced in patients with COVID-19 could be a primary cause of headache and general aches and pains that are associated with the disease (Fig. 1). Indeed, this may be the case, but not likely in all patients.^69,85^ Despite evidence of a robust type I interferon response in mild disease, several recent studies conducted in patients with severe COVID-19 have demonstrated that the virus seems capable of evading type I interferon induction^25,47,97^ and may be a key reason why a subset of patients are unable to mount an antiviral response.^4,69^ The lack of type I interferon induction may be due to antagonism of interferon signaling pathways by SARS-CoV-2 proteins, such as nsp6, nsp13, ORF3b, and ORF6.^37,97^ This evasion can lead to runaway viral replication and the systemic hyperinflammatory response, often termed cytokine storm, which is characteristic of severe COVID-19 disease.^4,55,107^ Another recent finding is that people with inborn errors in the type I interferon response or people who have autoantibodies to type I interferons are particularly susceptible to severe disease and mortality.^8,10,105^ It is increasingly clear that deficits in type I interferon responses of several kinds are key players in severe COVID-19.

An alternative mechanism of virus-elicited pain is through upregulation of IDO1 and increased production of kynurenine. In mice, viral infection increases IDO1 production in the spleen causing systemic increases in kynurenine.^32^ Kynurenine exposure in naive mice induced pain hypersensitivity, and viral infection-induced pain hypersensitivity was absent in mice lacking IDO1. In patients with COVID-19, one study found significant increases in kynurenine, kynurenic acid, picolinic acid, and nicotinic acid concentrations in patient's sera, indicative of kynurenine pathway activation.^83^ It is notable that a study in mice found that kynurenine signaling was associated with nerve injury-induced depression but not pain behaviors,^45^ thus whether altered kynurenine metabolism influences pain in COVID-19 remains unclear.

The ACE2 gene is known to be expressed in human skeletal muscle, thus direct damage to muscle tissue is also a possible cause of myalgia and widespread musculoskeletal pain in the acute phase of COVID-19 infection. The role of systemic inflammation in augmenting musculoskeletal pain, however, is also a likely contributing factor, particularly given the elevated levels of known nociceptive mediators, such as interleukin-6 (IL-6) in the sera of patients with COVID-19. Localized muscle inflammation has been observed in COVID-19, including a report of COVID-19–associated myositis, whereby myalgia coincided with proximal limb weakness and diffuse muscle edema with perivascular inflammatory infiltration.^103^ In other viral conditions, infected synovial macrophages are believed to propagate joint inflammation and arthralgia through the localized release of proinflammatory cytokines and matrix metalloproteinases.^74^ In muscle tissue collected postmortem from patients with SARS-CoV-1, widespread muscle fiber atrophy and immune cell infiltration was observed in several studies.^20,30,48^ Neuronal demyelination in SARS-CoV-1 infections was also noted, which may have contributed to muscle weakness and fatigue,^20^ although whether this will be seen in SARS-CoV-2 is currently unclear. It has been previously suggested that COVID-19, particularly severe infection associated with hyperinflammation, could act as a triggering factor for the development of autoimmune or autoinflammatory disorders.^14,106^ Although further research is clearly needed in this area, it is worth noting that after the outbreak of SARS-CoV-1, some patients went on to develop a myalgic encephalomyelitis/chronic fatigue syndrome (ME/CFS)-like illness that include symptoms of persistent fatigue and diffuse myalgia for up to 20 months.^62^ Although the pathogenesis of viral-attributed ME/CFS remains a topic of continued research, the high genetic similarity between SARS-CoV-1 and SARS-CoV-2 highlights the potential for similar postviral pain syndromes to emerge.

## 3. COVID-19 and cytokine interactions with nociceptors as a driver of pain

**Figure 2.**
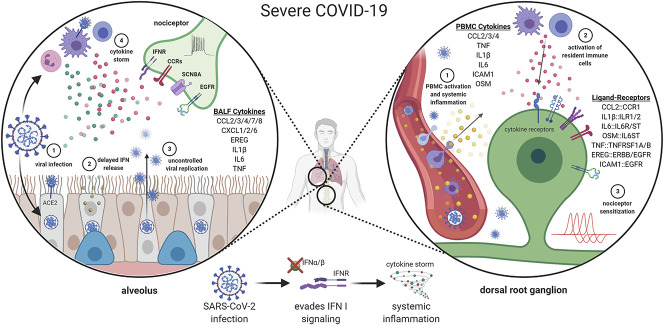
Potential mechanism(s) for nociceptor sensitization in severe COVID-19. SARS-CoV-2 enters lung epithelial cells and resident immune cells through ACE2. In severe COVID-19 cases, IFN production is abrogated allowing the virus to replicate unrestricted. Subsequent increase in viral load further enhances the production of inflammatory mediators and leads to the development of a hyperinflammatory state known as a “cytokine storm.” Cytokines produced in the lower respiratory tract may interact with receptors on sensory nerve endings promoting neurogenic inflammation and nociceptor hypersensitivity. An exaggerated immune response leads to systemic inflammation affecting multiple organs. As such, peripheral blood mononuclear cells (PBMCs) produce potent inflammatory mediators that have known receptors in the sensory neurons and resident immune cells of the DRG, driving nociceptor sensitization. ACE2, angiotensin-converting enzyme 2; DRG, dorsal root ganglia.

Similarly to other zoonotic coronaviruses, such as Middle East respiratory syndrome–related coronavirus and SARS-CoV-1, the SARS-CoV-2 virus has demonstrated a capacity for neurovirulence in humans.^62^ Despite a high prevalence of neurological complications being documented,^31,53^ the neurobiology and pathophysiology of SARS-CoV-2 remains unclear. In particular, how this might subsequently influence nociception (both acutely and in the longer term) is not yet known.^68^ To gain insights into how COVID-19 infection might cause pain-related conditions or worsen existing pain states, we have used our previously described interactome framework^91^ to critically reanalyze COVID-19 RNA-sequencing (RNA-seq) data sets for secretory ligands with known human DRG (hDRG) receptors. Through this ligand–receptor interactome, we are able to analyze any given gene list and identify those genes which (1) are capable (or expected to be capable) of paracrine effects and (2) have at least one known receptor on hDRG. Accordingly, this interactome allows us to extract genes with the potential to influence the human sensory nervous system. Using this approach, we have identified a number of transcriptional changes associated with COVID-19 infection with the potential to influence nociceptor sensitization (Fig. 2).

Given that acute respiratory distress syndrome is a significant and common complication of severe infection, a number of studies have performed RNA-seq of bronchoalveolar lavage fluid (BALF) as a means of characterizing the immune response in COVID-19. In the bulk RNA-seq of BALF, the expression of several proinflammatory mediators with known hDRG receptors seem upregulated after severe infection, including *CCL2/3/4/7/8* and *CXCL1/2/6*.^72,98^ In addition, the expression of the peptide hormone *EREG* and members from the ephrin family (*EFNA1* and *EFNA5*) were also upregulated in COVID-19 samples.^72,98^ Epiregulin (EREG) has been shown to sensitize nociceptors through epidermal growth factor family receptors,^54^ whereas ephrins are known to contribute to neuronal modulation.^27^ Similar to the bulk RNA-seq, our secondary analysis of single-cell RNA-seq (scRNA-seq) data sets also found significantly upregulated expression of proinflammatory mediators capable of interacting with hDRG neurons. Specifically, in scRNA-seq data sets, an increased abundance of monocyte-derived macrophages was associated with increased transcriptional expression of *CCL2/3/4* and *IL1B*, as well as several members of the tumor necrosis factor superfamily (*TNF, TNFSF10, TNFSF12, and TNFSF13B*), in severe infection compared with moderate COVID-19 and/or healthy controls.^26,49^ Importantly, the proteins encoded by these genes all have receptors in thoracic hDRG, and thus their increased expression in BALF may modulate sensory innervation in the lung. Differences between the cellular landscape in moderate and severe COVID-19 BALF samples suggest that localized inflammation in the lungs is driven by different cellular populations and associated with different cytokine and chemokine profiles.^49^ Importantly, this may mean that the varying severities of COVID-19 may contribute to local nociceptor sensitization or signaling within the lungs in discrete ways.

Bronchoalveolar lavage fluid samples are collected directly from the lungs, thus the extent to which locally released inflammatory mediators might elicit neuronal effects beyond the immediate lung parenchyma is unclear. Fortunately, there have also been several studies that characterize peripheral blood mononuclear cells (PBMCs) from patients with COVID-19 and thus allow us to explore the potential for broader effects of systemically released ligands using our interactome platform. In severe COVID-19, significant changes in relative immune cell proportions among PBMCs were seen in multiple scRNA-seq studies when compared with healthy controls, consistent with an extensive immune response.^46,86,92,104^ This included increases in classical monocytes, expansion of effector T-cell subsets, and decreases in plasmacytoid dendritic cells in severe disease.^46,92,104^ Similar to BALF findings, we found mild and severe COVID-19 PBMCs to exhibit transcriptional upregulation of prototypical signaling ligands that have known hDRG receptors, including *IL1B* and *TNF*. In addition to their effects on nociceptor sensitization, it has been previously reported that increased IL-1β and TNFα signaling can also increase the blood–brain barrier (BBB) permeability and facilitate the central nervous system (CNS) entry of neurotropic viruses.^60,89^ Both IL-1β and TNFα are upregulated at the protein level in severe COVID-19^25,31^; however, discrepancy between TNFα transcriptional regulation and associated plasma protein levels suggests that injured tissue (as opposed to circulating immune cells) may be the source of TNFα in severe infection.^25^ Similarly, IL-6 concentrations are often elevated in plasma despite a lack of transcriptional upregulation and an impaired cytokine response in blood myeloid cells and plasmacytoid dendritic cells.^4,25^ This again suggests that injured tissues such as lung and/or endothelia may be the cellular source of IL-6 in severe/critical infection. Given the propensity for systemic IL-1β, TNFα, and IL-6 to contribute to peripheral and central neuronal sensitization,^11,52,94^ it can be hypothesized that the increased prevalence of neurological manifestations seen in severe COVID-19 may be associated with elevations of these cytokines.

Although IL-1β, IL-6, and TNFα are well-established to influence nociceptive hypersensitivity, the possibility that additional or alternative mediators may also be driving nociceptive sensitivity in patients with COVID-19 cannot be discounted, especially in severe and critical infection. In several RNA-seq data sets, chemokine transcripts, including *CCL2/3/4* and *CXCL2,* were also upregulated in PBMCs from severe COVID-19 compared with healthy controls.^46^ These findings have also been confirmed at the protein level for CCL-2, CCL-3, and CCL-4 in the sera of patients with severe COVID-19.^25,99^ In addition to having known receptor targets on hDRG, both CCL-2 and CCL-3 also contribute to the development of nociceptor sensitization in rodents.^1,2^ Furthermore, increased circulating CCL-2 (also known as monocyte chemoattractant protein 1 [MCP-1]) has been shown to promote brain infiltration of monocyte-derived macrophages in viral encephalitis.^15^ Its receptor, CCR2, is also implicated in promoting CNS neurotropism during murine coronavirus infection.^15^ Increased CCL-3 (also known as macrophage inflammatory protein-1α [MIP-1α]) during murine coronavirus infection has similarly been associated with the differentiation and migration of effector T cells into the brain parenchyma,^84^ thus there is a potential for these cytokines to augment pain responses and facilitate neuroinflammation during COVID-19 infection. Further to these ligands, we also noted increased expression of *EREG*, *OSM, B2M*, as well as type II HLA molecules *HLA-A*, *HLA-B*, *HLA-C*, and *HLA-E* predominantly in CD4^+^ and CD8^+^ T cells and monocytes in patients with severe COVID-19. These cellular subsets were typically expanded in severe COVID-19 compared with healthy controls.^46,92,104^ In addition to the aforementioned role of EREG in pain processing, oncostatin-M (OSM) has previously been demonstrated to have a role in nociceptor sensitization.^22,43^ β2 Microglobulin (B2M) is associated with the heavy chain of major histocompatibility complex, class I (MHC-I). In TRPV1-expressing mouse DRG neurons, B2M was found to increase Erk phosphorylation, indicative of neuronal activation.^3^ Class I HLA alleles have been implicated in the development of postherpetic neuralgia, possibly through weak HLA binding affinity causing a suboptimal antiviral immune response.^59^ These mediators all have at least one known hDRG receptor, with some having previously been documented to sensitize rodent or human nociceptors.^22,43,54^

The validation of transcriptional changes at the protein level is an essential component of understanding the true mechanism(s) driving nociceptive plasticity in COVID-19. As might be expected, the plasma concentrations of prototypical inflammatory mediators TNFα, IL-1β, and IL-6 have been frequently reported in COVID-19 studies and seem to increase relative to infection severity in most patients.^25,31^ The concentrations of other ligands with known receptors in the hDRG have been less commonly reported. In one study, the plasma levels of OSM, a member of the IL-6 family, was found to strongly correlate with the clinical severity of COVID-19 infection.^4^ This pleiotropic cytokine is associated with neuropathic pain in patients,^67^ thus increased OSM in patients with severe COVID-19 could also contribute to increased susceptibility for acute pain and/or may worsen existing pain states. Furthermore, although CXCL2 (also known as macrophage inflammatory protein-2α [MIP-2α]) was transcriptionally upregulated in BALF samples, one study found no significant difference in plasma concentration between healthy controls and the various stratifications of COVID-19 infection severity, yet the CXCR2 was upregulated in severe disease.^25^

In addition to protein-coding transcripts, the impact of SARS-CoV-2 on microRNA (miRNA) and long noncoding RNA (lncRNA) warrants consideration. MicroRNAs are small noncoding RNAs that regulate gene expression by translational inhibition or mRNA degradation, whereas lncRNAs are capable of regulating miRNA function. It has been suggested that host-encoded miRNAs are capable of modulating viral infections as part of the human immune response.^23^ In the context of SARS-CoV-2, available data and predictive analyses suggest that infection-induced host miRNAs may inhibit some immune surveillance pathways, including IFN-γ signaling, TGF-β signaling, and toll-like receptors, all of which may contribute to immune suppression.^34^ The effect miRNAs might have on either disease course or pain processing is (as of yet) unclear. Similarly, although several differentially expressed lncRNAs have been identified in COVID-19 patient-derived lung tissue,^87^ their mechanistic role in viral infection and pain processing remain thus far poorly understood and highly speculative.

Alongside the dysregulated inflammatory response seen in COVID-19, hypercoagulopathy is another significant and defining feature of severe and critical disease.^28,82^ Hypercoagulopathy has been associated with an increased risk of stroke in severe COVID-19 and is likely related to immune dysregulation, although it may also be a result of sepsis.^28,53^ Indeed, one study found that the profiles of select cytokines in patients with severe COVID-19 (including IL-1β, IL-6, and TNFα) were similar to those found in patients with critically ill sepsis or acute respiratory distress syndrome.^93^ Given that the cytokine storm produced in severe COVID-19 resembles what is seen in sepsis, it can be hypothesized that neuropathological outcomes may be a consequence of surviving sepsis. There is surprisingly little work conducted on this topic, but the existing studies into neurological sequelae postcritical illness paint a dire picture. A study of 14 critically ill patients in Italy found that all patients had a loss of intraepidermal nerve fibers (IENFs) and close to half had clinical symptoms of small fiber neuropathy or dysautonomia.^44^ A follow-up study found that these changes in IENFs occur rapidly during the course of critical illness.^78^ A retrospective study performed in Australia examined chronic pain outcomes in critically ill patients and found that 44% of these patients developed chronic pain; sepsis was the biggest risk factor for chronic pain.^9^ Taken together, these studies suggest that an outcome of the cytokine storm seen in COVID-19 may be chronic pain. A better understanding of the mechanisms that might drive these cytokine-mediated interactions with nociceptors can help us be in a better position to treat the many thousands of new cases of chronic pain that will potentially result from the pandemic.

## 4. SARS-CoV-2 neurotropism and its implications for pain and/or neuropathy

There are many examples from other disease states, whereby viruses can interact directly with neurons.^38^ The varicella-zoster virus infects sensory neurons where it can sit dormant for decades before inducing a shingles attack. The rabies virus infects motor neurons and then uses these neurons as a gateway to the CNS. Thus, we must ask: does the SARS-CoV-2 have the ability to infect neurons, and, if so, which neurons might it target?

**Figure 3.**
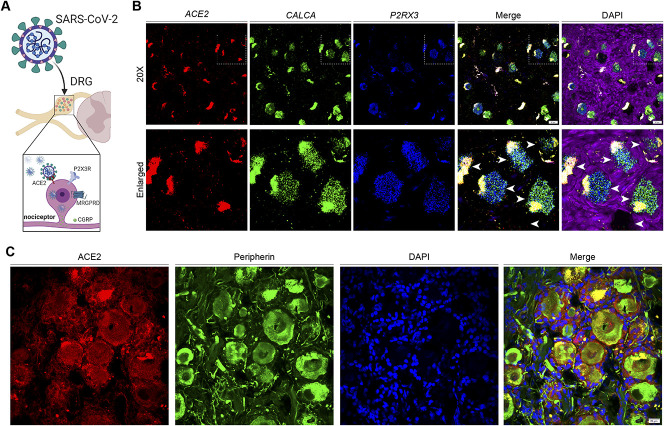
ACE2 expression in the dorsal root ganglia. (A) The ability of SARS-CoV-2 to directly target sensory neurons in the DRG depends on the expression of ACE2. (B) We recently demonstrated that ACE2 mRNA is expressed by *P2X3R*, *CALCA*, and *MRGPRD* expressing neurons. White arrowheads indicate ACE2-positive neurons in the human DRG. Lipofuscin in human DRG neurons appears white in the merged images. Although *ACE2* mRNA is present in 20% of human DRG neurons, the expression level is low. (C) Interestingly, ACE2 immunoreactivity in human DRG is diffuse, suggesting that the extracellular protein may contact many cell types. ACE2, angiotensin-converting enzyme 2; CGRP, calcitonin gene-related peptide; DRG, dorsal root ganglia.

At the transcriptional level, a subset of human nociceptors that express the *MRGPRD* and *CALCA* genes also express *ACE2* mRNA. This suggests that neurons that innervate the outer most layers of the skin^108^ and the lining of hollow organs^29^ likely express the ACE2 receptor. Interestingly, in mice, neurons that express some of these markers innervate the meninges.^88^ A neurological hallmark of COVID-19 is headache.^53,63^ Because headache pain is driven by nociceptors that innervate the meninges,^33,35^ this implies that SARS-CoV-2 infection of these neurons may be a cause of this symptom. Using 2 independent antibodies for ACE2 protein, we have observed immunoreactivity in hDRG neurons, consistent with the expression of functional ACE2 receptors by these neurons (Fig. 3A, B).^77^

Khanna et al. recently reported that the SARS-CoV-2 spike protein interacts with NRP1 to induce signaling in nociceptors that then causes antinociceptive effects.^64^ Neuropilin 1 is strongly expressed in hDRG^71,90^ and is found in rodent nociceptors.^64^ Importantly, NRP1 was recently reported as a host entry receptor for SARS-CoV-2.^13,19^ This happens because when the spike protein is cleaved by the protease furin, which is also expressed in hDRG,^76^ the processed protein has a high affinity for NRP1 and NRP2. Two recent reports demonstrate that the expression of ACE2, furin, and NRP1 or NRP2 augments SARS-CoV-2 cellular infection and that this is a likely mechanism through which SARS-CoV-2 infects the olfactory epithelium.^13,19^ Because ACE2 is a secreted protein that shows diffuse staining in hDRG (Fig. 3C) and furin and NRP1 are likely expressed by many nociceptors,^64,76^ this increases the probability that the virus may infect these cells. Our view is that it will be very important to understand the consequences of spike protein and SARS-CoV-2 interactions with human nociceptors as this will inform our understanding of the long-term effects of the virus on these neurons.

Studies in human brain organoids have shown neurotropic effects of SARS-CoV-2. In this model system, SARS-CoV-2 infection of human neurons was completely dependent on ACE2 expression and did not induce cell death to infected neurons but did cause severe detrimental effects to surrounding cells.^80^ Using an ACE2 overexpression model in mice, these authors demonstrated that neuronal ACE2 overexpression is associated with increased mortality from SARS-CoV-2 infection, suggesting clinically relevant effects of neurotropism in COVID-19.^80^ It is currently unclear, however, whether there is brain penetration by SARS-CoV-2, with 18 consecutive brain autopsies of patients with PCR-confirmed COVID-19 finding no neuronal infection.^79^ Moreover, a study using single-cell sequencing of BBB and cortical neurons found viral infection in the BBB and choroid plexus but no clear evidence of viral infection in cortical neurons.^101^ This study did, however, clearly demonstrate that BBB infection can lead to T-cell invasion in the cortex and signs of microglial activation to a phagocytotic phenotype that may have a profound impact on cortical neuron function.^101^ Therefore, the BBB may both protect the brain from direct SARS-CoV-2 infection, but on infection of BBB cells, serve as an entry point for inflammatory cells that then cause pathological changes in the brain.

More work will clearly be needed to understand whether SARS-CoV-2 infects nociceptors, but the existing evidence suggests this is possible, if not likely. It will be important to understand the consequences of this nociceptor neurotropism and whether it can cause long-term effects that might lead to or worsen neuropathies. Unlike herpes viruses, there is no precedent for coronavirus latency in neurons or other cell types so a shingles-like disease emerging years after COVID-19 is unlikely.^38^

Some concerns have been raised regarding the potential for pharmacologically induced changes to ACE2 expression,^21^ which could have an effect on the neurotropism of SARS-CoV-2. Animal studies have previously found that ibuprofen upregulated ACE2 protein in diabetic rats,^70^ whereas drugs such as ACE inhibitors and angiotensin II type-I receptor blockers had previously been associated with downregulation of ACE2.^96^ Upregulation of ACE2 may facilitate host infection, providing an increased cell entry point for SARS-CoV-2. Conversely, it has also been suggested that upregulation of ACE2 may provide pulmonoprotective effects against virus-induced lung damage through reducing binding of angiotensin II to the AT1 receptor and subsequent downstream production of effectors, such as TGF-β1.^40^ Although clinical implications of these in vivo findings caused confusion and trepidation in early stages of the COVID-19 pandemic, recent data from patients with COVID-19 show that non-steroidal anti-inflammatory drugs use is not associated with 30-day mortality, hospitalization, intensive care unit admission, mechanical ventilation, or renal replacement therapy in patients with COVID-19.^12,50^ Similarly, a systematic review found that ACE inhibitors or angiotensin II type-I receptor blockers use was not associated with an increased severity of COVID-19 illness.^51^

## 5. Conclusions

Since its emergence in late 2019, extensive research into the SARS-CoV-2 and its clinical presentation has identified a number of neuropathologies associated with this pathogen, including pain-related conditions. Although there have been no dedicated mechanistic studies thus far looking at how the SARS-CoV-2 virus might directly impact nociceptive hypersensitivity, the extensive, systemic hyperinflammation seen in severe COVID-19 has the potential to contribute to nociceptor sensitization. Through secondary analyses of publicly available RNA-seq data sets, we have identified several secreted ligands that have the potential to modulate sensory neurons in humans after severe COVID-19 infection. It remains unclear, however, whether the mechanism(s) of neuronal sensitization in severe COVID-19 are the same as those in milder cases, given that both the nature and extent of immune response in COVID-19 can be significantly different depending on infection severity. The impact of cytokine storm must also be considered as a potential driving factor for the development of neuropathies after severe infection and could contribute to the development of chronic pain after acute COVID-19 infection has resolved. Continued research into the mechanisms of SARS-CoV-2 neurovirulence, including whether direct neuronal infection is possible, will be essential to not only help us understand how this pathogen acutely impacts neuronal hypersensitivity but also whether this is dictated by infection severity, and how these neuronal changes might contribute to COVID-19 survivors experiencing painful sequelae in the long term.

## Disclosures

The authors have no conflicts of interest to declare.

This work was supported by NIH grants NS065926 and NS111929 to T.J. Price. T.J. Price is a co-founder of 4E Therapeutics, a company developing MNK inhibitors for neuropathic pain.
